# SARS-CoV-2 Infection Precipitates the Discovery of Underlying Liver Disease: A Case Report

**DOI:** 10.7759/cureus.37811

**Published:** 2023-04-19

**Authors:** Victoria Diaz, Elizabeth Benge, Matthew Brockway, Weston Truman, Birjees Ahmed

**Affiliations:** 1 Internal Medicine, Mountainview Hospital, Las Vegas, USA; 2 Internal Medicine, MountainView Hospital, Las Vegas, USA

**Keywords:** gastroenterology and hepatology, hepatology, a1at, transaminitis, covid-19, alpha-1-antitrypsin deficiency

## Abstract

Since the onset of the severe acute respiratory syndrome coronavirus 2 (SARS-CoV-2) pandemic, numerous sequelae of this devastating virus have come to light. One organ known to be impacted by SARS-CoV-2 is the liver, as many SARS-CoV-2 patients demonstrate elevated liver enzymes on routine laboratory tests. In this case report, we present a patient with SARS-CoV-2 whose liver enzymes remained persistently elevated throughout his hospitalization. Due to the duration of his elevated liver enzymes, etiologies outside of SARS-CoV-2 were explored. This workup revealed that the patient had alpha-1 antitrypsin (A1AT) deficiency. Thus, this case serves to remind clinicians to continue investigating lab abnormalities despite a presumed etiology, such as SARS-CoV-2, so as not to miss the presentation of new diagnoses.

## Introduction

The world has experienced the economic, physical, and emotional devastation of severe acute respiratory syndrome coronavirus 2 (SARS-CoV-2). As of April 2023, the Centers for Disease Control and Prevention (CDC) reports that there have been over 104,348,746 cases and over 1,128,404 deaths in the United States of America [1].

As more is learned about SARS-CoV-2, leading experts note the trend of pulmonary as well as extrapulmonary consequences, including thrombotic events such as acute coronary syndrome, acute kidney injury, vascular sequelae, and liver injury [2]. To date, researchers have found that SARS-CoV-2 can injure the liver in multiple ways. These injuries include hypoxia from respiratory failure, coagulopathy, direct cellular injury from infection, drug-induced injury, and exacerbation of underlying liver pathology like alpha-1 antitrypsin (A1AT) deficiency [3]. A1AT deficiency is a congenital disease that causes altered production of this protease inhibitor enzyme, which accumulates in hepatocytes causing hepatocellular injury and decreased inhibition of neutrophil elastase that, when left unopposed, causes destruction of alveoli [4].

Herein, we present the case of a man who contracted SARS-CoV-2, developed a hepatic injury, and was found to have an underlying heterozygous (MZ) phenotype of A1AT deficiency. The presented article follows the CARE reporting checklist.

## Case presentation

A previously healthy 57-year-old male patient with a past medical history of exercise-induced asthma presented to the emergency room with progressively worsening shortness of breath. His only home medication was an as-needed albuterol inhaler to treat asthma symptoms. On presentation, he noted the more frequent use of his inhaler due to the recent worsening of his asthma symptoms. He also endorsed three months of fatigue. The patient consumed two to three servings of alcohol weekly and denied other tobacco or substance use. His family history was unknown. SARS-CoV-2 vaccines were not available at the time, therefore the patient was unvaccinated. A physical exam revealed bilaterally decreased breath sounds with diffuse wheezing.

The patient presented to our emergency room in December 2020 and tested positive for SARS-CoV-2 on the rapid antigen test. Initial laboratory results were significant for an elevated white blood cell count of 16,000 and mildly elevated liver enzymes: aspartate aminotransferase (AST) of 90 and alanine aminotransferase (ALT) of 124 (Table 1). The patient had routine laboratory blood work performed with his primary care physician just three months prior to presentation, and his liver enzymes were within normal limits at that time. Abdominal ultrasound showed fatty liver infiltration. Computed tomography (CT) scans of the chest were suggestive of liver cirrhosis in the inferior slices; however, the patient was discharged before CT of the abdomen was done.

**Table 1:** Laboratory values on admission

Laboratory	Value	Reference range
White blood count (10^3/μL)	16	4.8-10.8
Hemoglobin (GM/dL)	18	14.0-18.0
Total bilirubin (mg/dL)	1.1	0.1-1.1
Aspartate aminotransferase (U/L)	90	10-41
Alanine aminotransferase (U/L)	124	12-78
Alkaline phosphatase (U/L)	238	98-317
Alpha-1 antitrypsin (mg/dL)	35	100-220

The patient’s newly elevated liver enzymes, in conjunction with his finding of liver cirrhosis, prompted further workup. Iron studies showed elevated iron levels but were normal for transferrin, saturation, and ferritin. Negative tests included hemochromatosis mutation, heparin-induced thrombocytopenia (HIT) and urine drug screen. Ceruloplasmin and both serum and urine copper studies were within normal limits. Viral studies were negative for hepatitis A (HAV), B (HBV), and C (HCV), as well as human immunodeficiency virus (HIV). The patient was found to have a low alpha-1 antitrypsin (A1AT) level of only 35 mg/dL. The patient was positive for the MZ genotype, therefore he was heterozygous for a Z-mutation (a deficiency allele) in the SERPINA1 gene (a gene coding instructions for the production of A1AT) causing significant decreases of A1AT. 

By the time A1AT results were obtained, the patient's SARS-CoV-2 symptoms had improved and the patient was discharged from the hospital in stable condition. He declined intravenous augmentation therapy with A1AT during hospitalization due to healthcare costs and stated he would follow up outpatient with his primary care physician but was lost to further follow-up.

## Discussion

As more is divulged about the nature of SARS-CoV-2 and its effects on the respiratory system, so too have its effects on other organ systems. Data reports that the hepatic system is commonly affected by SARS-CoV-2; 14% to 76% of patients suffering from SARS-CoV-2-related pneumonia are also found to have elevated liver enzymes [5,6]. Similarly, A1AT deficiency patients have both respiratory and hepatic manifestations. SARS-CoV-2 patients with elevations in aspartate aminotransferase (AST) have been shown to have a higher risk of mortality [5]. A1AT deficiency causes hepatocytes to house mutant Z (deficiency allele) protein polymers that trigger apoptotic death, as does the entry of SARS-CoV-2 into hepatocytes; this compounded hepatocyte damage leads to the increased leakage of transaminases during times of stress [7].

In this case, we discuss a previously healthy patient who presented both positive for SARS-CoV-2 and with elevated liver enzymes. Of note, the patient’s liver enzymes were within normal limits only three months earlier on routine blood studies with his primary care provider. Thus, the initial leading differential suspected the elevated liver enzymes were secondary to that of the acute SARS-CoV-2 infection, given that it is known that SARS-CoV-2 can induce apoptotic processes in hepatocytes elevating liver enzymes [7]. It has been hypothesized that SARS-CoV-2 may utilize host angiotensin-converting enzyme 2 (ACE2) receptors, expressed on alveolar cells (type I and II), as well as on hepatocytes, both of which are the cells primarily affected in those with A1AT deficiency [8].

**Figure 1:**
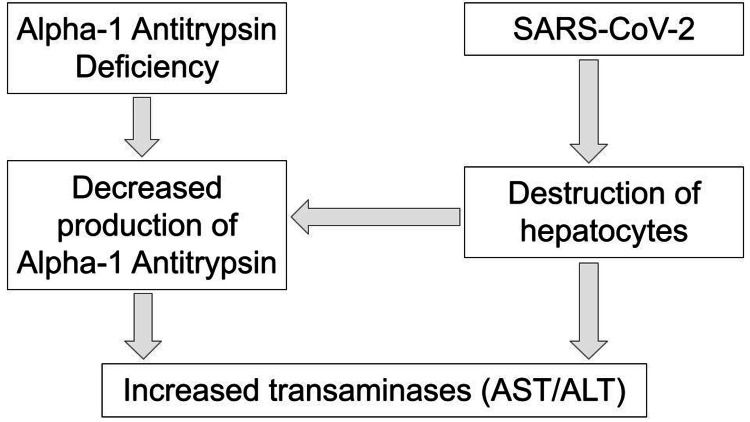
Pathophysiology of increased transaminases seen in A1AT deficiency and SARS-CoV-2 A1AT: alpha-1 antitrypsin; SARS-CoV-2: severe acute respiratory syndrome coronavirus 2

The effects of SARS-CoV-2 on our patient’s hepatocytes, which were already unknowingly under stress from an undiagnosed A1AT deficiency, explain our patient’s elevated liver enzymes found on admission. This prompted further investigation, leading to the discovery of his diagnosis. SARS-CoV-2 can exacerbate underlying chronic liver disease, which subsequently leads to higher mortality due to hepatic decompensation and acute-on-chronic liver failure [8]. Once we ruled out toxic and viral causes for elevated liver enzymes, genetic etiologies were next on our diagnostic path. Computed tomography revealed areas of liver cirrhosis and the challenge of explaining these structural changes presented, leading to suspicion of other underlying causes of liver disease becoming differentials. Around 10% of adults diagnosed with A1AT deficiency will present with liver cirrhosis [9]. Further investigation found the patient to have heterozygosity of a Z-mutation on the SERPINA1 gene causing the significant decreases in A1AT, leading to a definitive diagnosis and an explanation for the patient’s elevated liver enzymes. This case highlights the damaging effects of SARS-CoV-2 on hepatocytes, and the extensive synergistic destruction caused when infecting a patient with underlying liver pathology (Figure 1).

## Conclusions

When treating patients with SARS-CoV-2 infection, providers should understand the multisystem effects of SARS-CoV-2. This vast array of diverse SARS-CoV-2 manifestations will need to be further studied, especially as we see the emergence of new strains that have differing affinities for various systems. Our patient’s presentation demonstrated how the diagnosis of his underlying liver disease was incidentally detected because of the additional stress and damage his coexisting SARS-CoV-2 infection was putting on his hepatic system. In conclusion, elevated liver enzymes in the setting of SARS-CoV-2 may be more than a known sequela, and it is important to rule out the possibility of other coexisting undiagnosed differentials.
